# Egestabase – An online evidence platform to discover and explore options to recover plant nutrients from human excreta and domestic wastewater for reuse in agriculture

**DOI:** 10.1016/j.mex.2024.102774

**Published:** 2024-05-25

**Authors:** Robin Harder, Geneviève S. Metson, Biljana Macura, Solveig Johannesdottir, Rosanne Wielemaker, Dan Seddon, Emma Lundin, Abdulhamid Aliahmad, Erik Kärrman, Jennifer R. McConville

**Affiliations:** a Environmental Engineering Group, Department of Energy and Technology, Swedish University of Agricultural Sciences, SE-756 51 Uppsala, Sweden; b Ecological and Environmental Modelling, Department of Physics, Chemistry and Biology, Linköping University, SE-581 83 Linköping, Sweden; c Department of Geography and Environment, Western University, London ON N6A 5C2, Canada; d Stockholm Environment Institute, HQ, SE-104 51 Stockholm, Sweden; e RISE Research Institutes of Sweden, SE-756 51 Uppsala, Sweden; f Eawag, Swiss Federal Institute of Aquatic Science and Technology, CH-8600 Dübendorf, Switzerland; g Nature-based Solutions Initiative, Departments of Biology and Geography, University of Oxford, Oxford OX1 3SZ, United Kingdom; h RISE Research Institutes of Sweden, SE-412 58 Göteborg, Sweden; i RISE Research Institutes of Sweden, SE-114 28 Stockholm, Sweden

**Keywords:** Egestabase, Nutrient circularity, Systematic map, Evidence synthesis, Ecotechnologies, Resource-oriented sanitation

## Abstract

Restoring nutrient circularity across scales is important for ecosystem integrity as well as nutrient and food security. As such, research and development of technologies to recover plant nutrients from various organic residues has intensified. Yet, this emerging field is diverse and difficult to navigate, especially for newcomers. As an increasing number of actors search for circular solutions to nutrient management, there is a need to simplify access to the latest knowledge. Since the majority of nutrients entering urban areas end up in human excreta, we have chosen to focus on human excreta and domestic wastewater. Through systematic mapping with stakeholder engagement, we compiled and consolidated available evidence from research and practice. In this paper, we present ‘Egestabase’ – a carefully curated open-access online evidence platform that presents this evidence base in a systematic and accessible manner. We hope that this online evidence platform helps a variety of actors to navigate evidence on circular nutrient solutions for human excreta and domestic wastewater with ease and keep track of new findings.

## Review Highlights

• Egestabase is a goldmine of evidence from research and practice on the recovery and reuse of plant nutrients found in human excreta and domestic wastewater.
• Egestabase is based on a coherent conceptual framework and enables searching with standardized terminology.
• This enables users to explore the option space and quickly navigate and find associated literature.

## Graphical abstract

**Figure:**
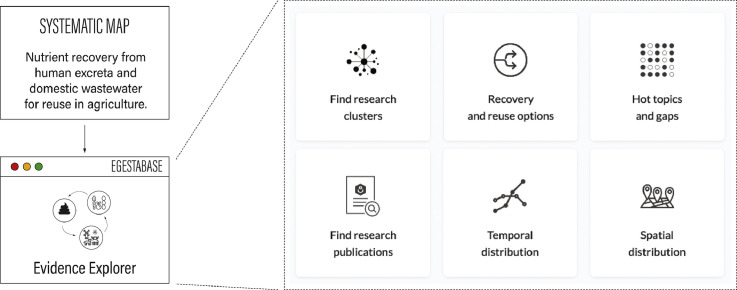

Specifications table

**Table:** 

Subject area:	Environmental Sciences
More specific subject area:	Nutrient recovery and reuse from human excreta and domestic wastewater
Name of the reviewed methodology:	Egestabase
Keywords:	online evidence platform, nutrient recovery and reuse, human excreta, domestic and municipal wastewater
Resource availability:	https://www.egestabase.net
Review question:	What evidence exists on technologies for the recovery of plant nutrients from human excreta and domestic wastewater for reuse in agriculture?

## Background

The importance of restoring nutrient circularity across scales is increasingly being understood, as evidenced by reviews of nutrient stock and flow analysis at the local scale (e.g., [1]) and studies that propose new conceptual frameworks for assessing nutrient circularity (e.g., [2,3]). Nutrient circularity on the one side is about recovering nutrients from organic residues for reuse in agricultural production. On the other side, it is about minimizing nutrient losses to the environment and thus implies a reduction of nutrient inputs to agricultural production. Improved nutrient circularity is closely linked not only to ecosystem integrity (notably by reducing nutrient pollution in water bodies), but also nutrient and food security (notably by reducing the dependency on finite agricultural inputs). Previous research has focused for instance on sustainable management of nitrogen (e.g., [4]) and phosphorus (e.g., [5]), or on the connection between nutrient management and food loops (e.g., [6]) or human excreta management (e.g., [7]). Organic residues suitable for nutrient recovery include crop and food residuals, animal and human manure, as well as streams that contain these organic residues. The focus of the work reported here is on human excreta and domestic wastewater – the organic residues where the majority of nutrients entering urban areas end up.

Research and development of technologies to recover plant nutrients from human excreta and domestic wastewater has intensified, as evidenced by the variety of technologies described in broad reviews of the topic (e.g., [8,9]), as well as the investigation of aspects regarding policies, markets and governance (e.g., [10]) or knowledge evolution (e.g., [11]). But navigating evidence from research and practice and keeping track of new findings is not a trivial task because of several challenges. First, evidence is scattered across different sources and primary research is often not readily collated and synthesized. Second, terminology is not standardized, making it difficult to create precise searches for evidence. For instance, similar concepts are referred to with different terms (the creativity of some researchers to coin new terms is sometimes astounding, and so is the sometimes sloppy use of terminology). Moreover, some terms are common across multiple scientific fields and terminology used in the context of nutrient recovery from human excreta and domestic wastewater is also used in other domains (e.g. blood urea *nitrogen* and *urine* output as biomarkers of renal *recovery* after acute kidney injury). Third, once potentially relevant evidence is found, it is not always easy to quickly extract information on what exactly is being reported. Taken together, these challenges make finding, synthesizing, and using evidence from research and practice a cumbersome and time-consuming process.

Imagine what it would be like to swiftly discover and explore various options for the recovery and reuse of nutrients found in human excreta and domestic wastewater – without the challenges associated with ambiguous terminology and diverse conceptual frameworks. Imagine there was a carefully curated knowledge base that compiles and consolidates available scientific evidence in a systematic and easily accessible manner – with standardized terminology based on a coherent conceptual framework. Over the past three years, under the umbrella of the research project *End-of-wastewater*, we made an attempt to do just this. This resulted in the *Egestabase*^1^ online evidence platform (available at www.egestabase.net) for navigating circular nutrient solutions.

The main novelty of Egestabase compared to more traditional bibliographic sources like Scopus and Web of Science is that it is geared towards a very specific knowledge domain – nutrient recovery and reuse from human excreta and domestic wastewater. This way, literature searches can be performed based on an explicit conceptual framework and standardized terminology for searches. Additionally, Egestabase features a visual representation of nutrient recovery and reuse options that can be used to interactively discover and explore different circular nutrient solutions. Similar efforts have previously been undertaken in the field of nature-based solutions, both regarding mapping [12,13] and the development of online knowledge bases [[14], [15], [16]]. This review broadly describes the process applied to develop Egestabase, as well as its functionality. We believe that Egestabase can make valuable contributions to obtaining a quick overview of the field of circular nutrient solutions, and to streamline future reviews and syntheses of selected subtopics of circular nutrient solutions covered in Egestabase.

## Method details

### Scope of the Egestabase evidence platform

**Fig. 1:**
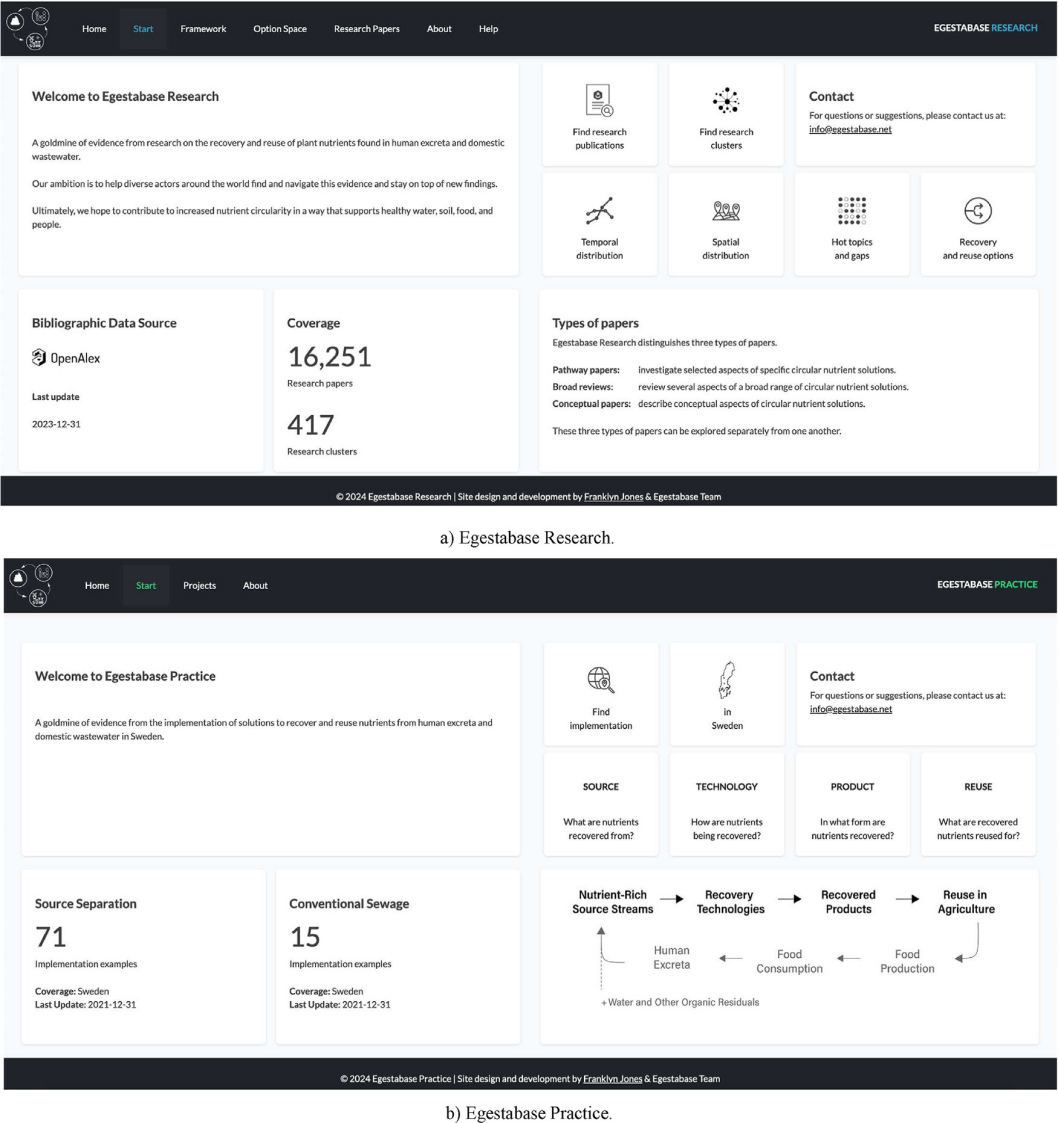
Egestabase landing pages.

Our aim with Egestabase was to facilitate the swift discovery of research publications (Egestabase Research) and implementation projects (Egestabase Practice). Egestabase Research currently comprises over 16 000 research articles (published between 1871 and 2023) and is designed such that users can easily explore temporal and spatial distributions of research evidence, hot topics and evidence gaps (i.e., research areas with much or little research), as well as different technological options for recovery and reuse (Fig. 1a). Egestabase Practice currently comprises over 80 implementation projects from Sweden (between 1967 and 2021) and is designed such that users can easily explore the type and spatial distribution of implementation projects (Fig. 1b).

### Conceptual framework

**Fig. 2:**
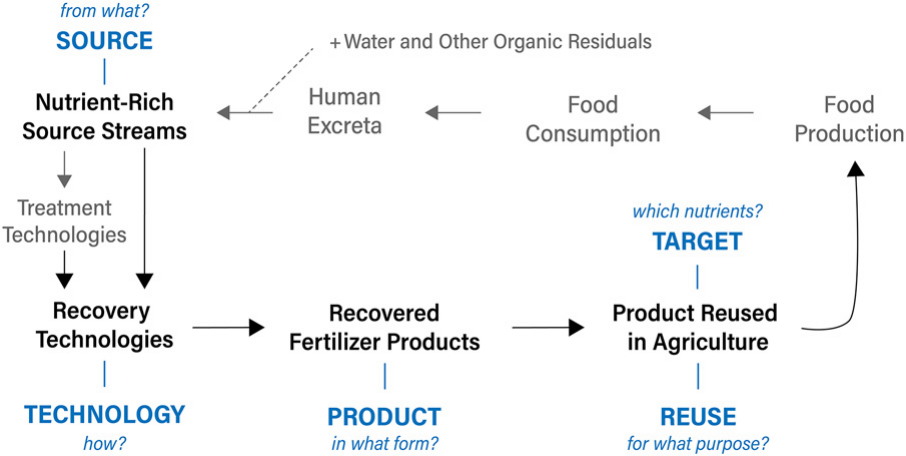
Conceptualization of nutrients flowing through food systems. Black arrows represent the scope of flows covered. Blue text in capital letters represents the recovery pathway coding applied in this article.

To categorize various approaches to the recovery of nutrients from human excreta and domestic wastewater, we chose five dimensions that together constitute a recovery pathway (Fig. 2). The recovery pathway describes which nutrients are being recovered, from what, how, in what form, and for what reuse purpose. Any given recovery pathway begins with an organic residue that is being managed - i.e., **source** stream (e.g., urine, domestic wastewater, sewage sludge ash). Following the collection and transportation of a given source stream, one or several recovery **technologies** (e.g., precipitation, leaching followed by precipitation) may be applied to recover nutrients. Recovery **targets** one or several critical plant nutrients (e.g., nitrogen and phosphorus) and results in a recovered fertilizer **product** (e.g., struvite) that can be **reused** back in agriculture and food production (e.g., as fertilizer, animal feed, or raw material for protein production).

The concept of recovery pathways was applied in analysis of both implementation projects and research papers that describe selected aspects of one or several technologies that recover nutrients from human excreta and domestic wastewater. For implementation projects, we also specified the type, scale, and context of the project. For research papers, we also specified the **domain** of a given research – that is, a focus on the collection system, technology development, product characteristics, the use of products in agriculture, or user acceptance.

In addition, we also identified conceptual papers that broadly describe important concepts and perspectives of relevance for restoring nutrient circularity. These papers we categorized in terms of broad concepts, such as ‘paradigm shift’, ‘circular nutrient economy’, ‘waste-as-resource’ or similar.

### Developing Egestabase

**Fig. 3:**
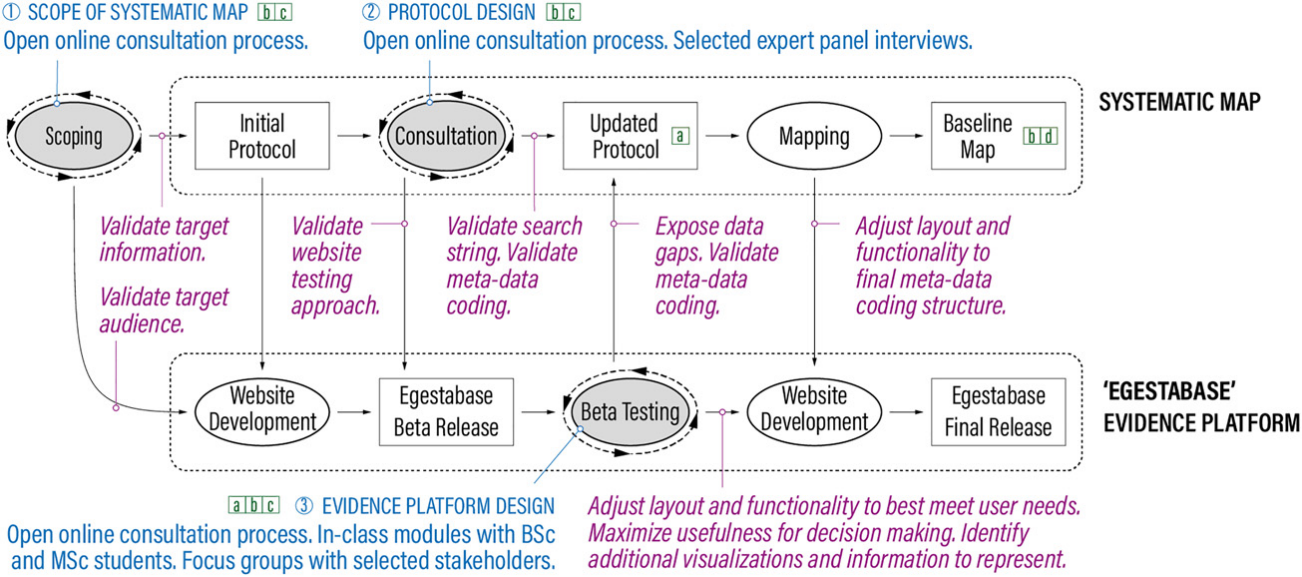
Overview of successive steps in developing the ‘Egestabase’ online evidence platform (adapted from [20]). Ovals represent processes and squares are resulting products. Co-design processes are highlighted in gray color. The associated text in blue color denotes types of interaction with stakeholders. Italicized text in magenta describes types of expected input to the design process. Green square boxes indicate previous papers that describe further details – a: Macura et al. [20]. b: Macura et al. [19]. c: Lundin et al. [18]. d: Johannesdottir and Harder [21].

***Co-design andstakeholderengagement.*** To ensure the relevance of the outcomes for different types of actors and encourage better evidence uptake into policy and practice [17], we used a co-design process with repeated stakeholder input throughout the development of Egestabase (see Fig. 3). Among others, we solicited stakeholder input to help us better understand the needs of actors who benefit from knowledge on nutrient recovery and reuse. We also beta-tested successive releases of Egestabase. Stakeholders that we consulted include representatives of academia (students, faculty, and researchers), utilities, and government agencies. Engagement was done through a combination of three methods: online focus groups, in-class activities, and open feedback processes. More details are provided in Lundin et al. [18] and Macura et al. [19].

***Mappingevidence from*** p***ractice.*** Evidence from practice in Sweden was collected through internet searches in English and Swedish and by contacting the 290 Swedish municipalities and consulting their urban water and sanitation planning documents. This yielded 86 implementation projects. More details on the case studies and the validation and limitations of the procedure are provided in Johannesdottir and Harder [21]. Evidence from practice in other countries was beyond the scope of our research project; besides much increased resources, it would also have required knowledge of local contexts and languages that we did not have.

***Mappingevidence fromresearch.*** Evidence from research was collected through searches on Scopus and Web of Science using various combinations of the keywords listed in Table 1 and further detailed in Macura et al. [20]. This search strategy was rather broad and yielded over 155 000 unique records, of which roughly 18 000 (11.5%) were considered relevant. More details on the evidence base, including the validation of the mapping process and limitations of the evidence base, are provided in and Macura et al. [19]. Technical details on retrieving and processing bibliographic records are reported in Harder [22]. Note that only research works indexed on OpenAlex (roughly 16 000 records) are included in Egestabase.

**Table 1:** List of search terms used to find relevant scientific literature (British English spelling).

Source	sanitation, watsan, ecosan, toilet, latrine, urinal, urine, feces, feces, excreta, excrement, human waste, human manure, humanure, night soil, yellowwater, brownwater, blackwater, fecal sludge, septage, sewage, sewerage, wastewater, digestate, effluent, sludge, slurry, biosolid
Product	fertiliser, conditioner, biomass, biosolid, char, compost, ash, algae, microalgae, duckweed, struvite, vivianite, calcium phosphate, hydroxyapatite
Reuse	recover, recycle, circulate, capture, valorise, utilize, reclaim, convert, return, reuse, fertilize, fertigate, irrigate, agriculture, feed, amendment, land apply
Product	nutrient, nitrogen, urea, ammonia, ammonium, phosphorus, phosphate, phosphoric, potassium, potash

***Website development.*** Website development took place over five iterations. The second and third iterations considered feedback from preceding stakeholder engagement. The fourth and fifth iteration resulted from changes in meta-data coding structure.

### Functionality of Egestabase

There are three main sections that make up Egestabase Research: ‘Framework’, ‘Option Space’, and ‘Research Papers’. There is one main section in Egestabse Practice: ‘Projects’.

### Egestabase Research

**Fig. 4:**
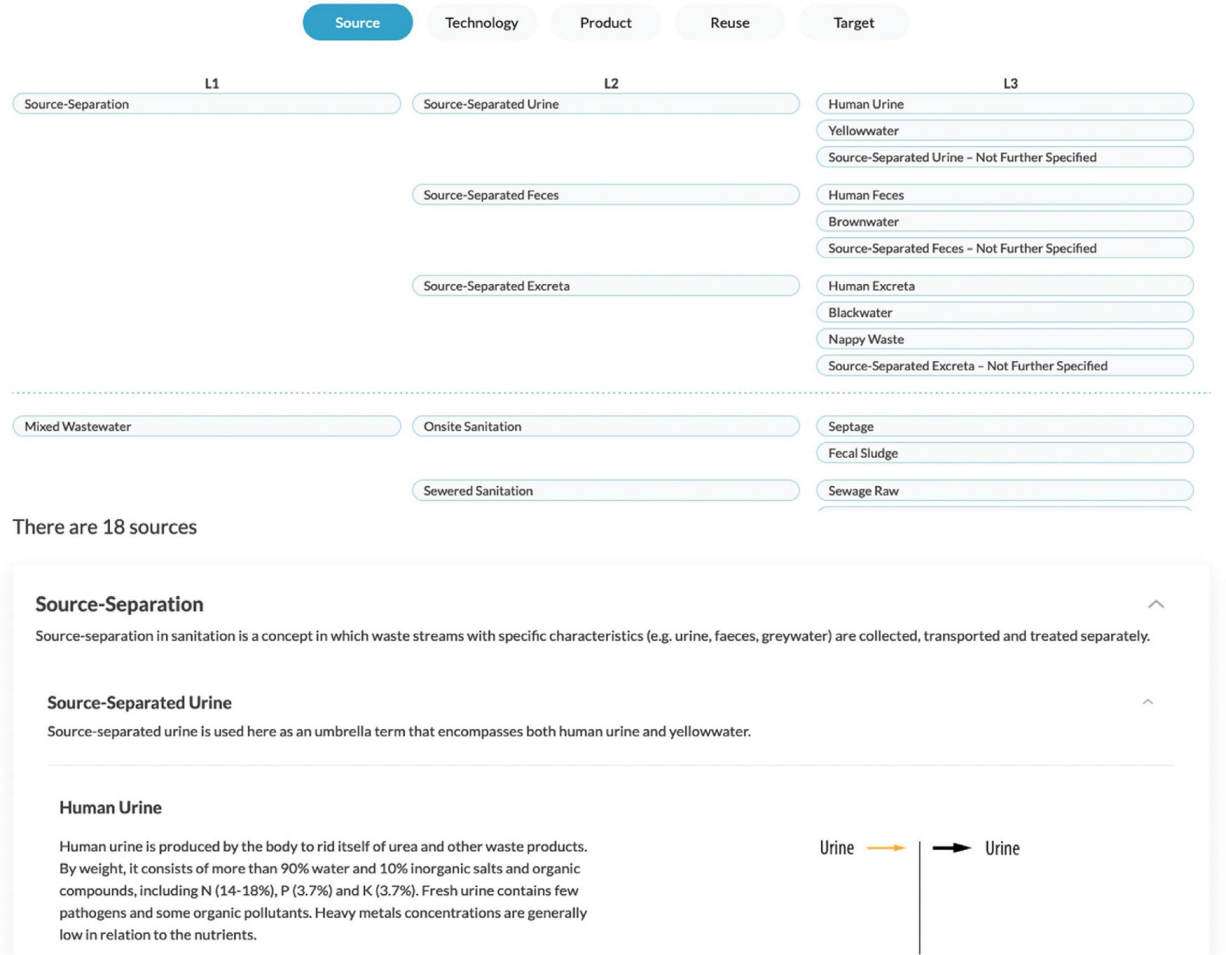
‘Framework’ section – with activated selection for ‘source’ dimension of recovery pathway. *Upper part*: hierarchy levels of the conceptual framework. *Lower part*: definition of terminology used.

***Framework.*** This section outlines the conceptual framework we devised and defines the terminology we used. For each recovery pathway dimension (i.e., Source – Technology – Product – Reuse – Target), we distinguish a hierarchy of three levels (see example in Fig. 4). This hierarchy allows users to navigate the dataset using different aggregation levels. In addition, we provide a definition for each element (e.g. Source-Separated Urine) that constitutes the option space (see example in Fig. 4).

**Fig. 5:**
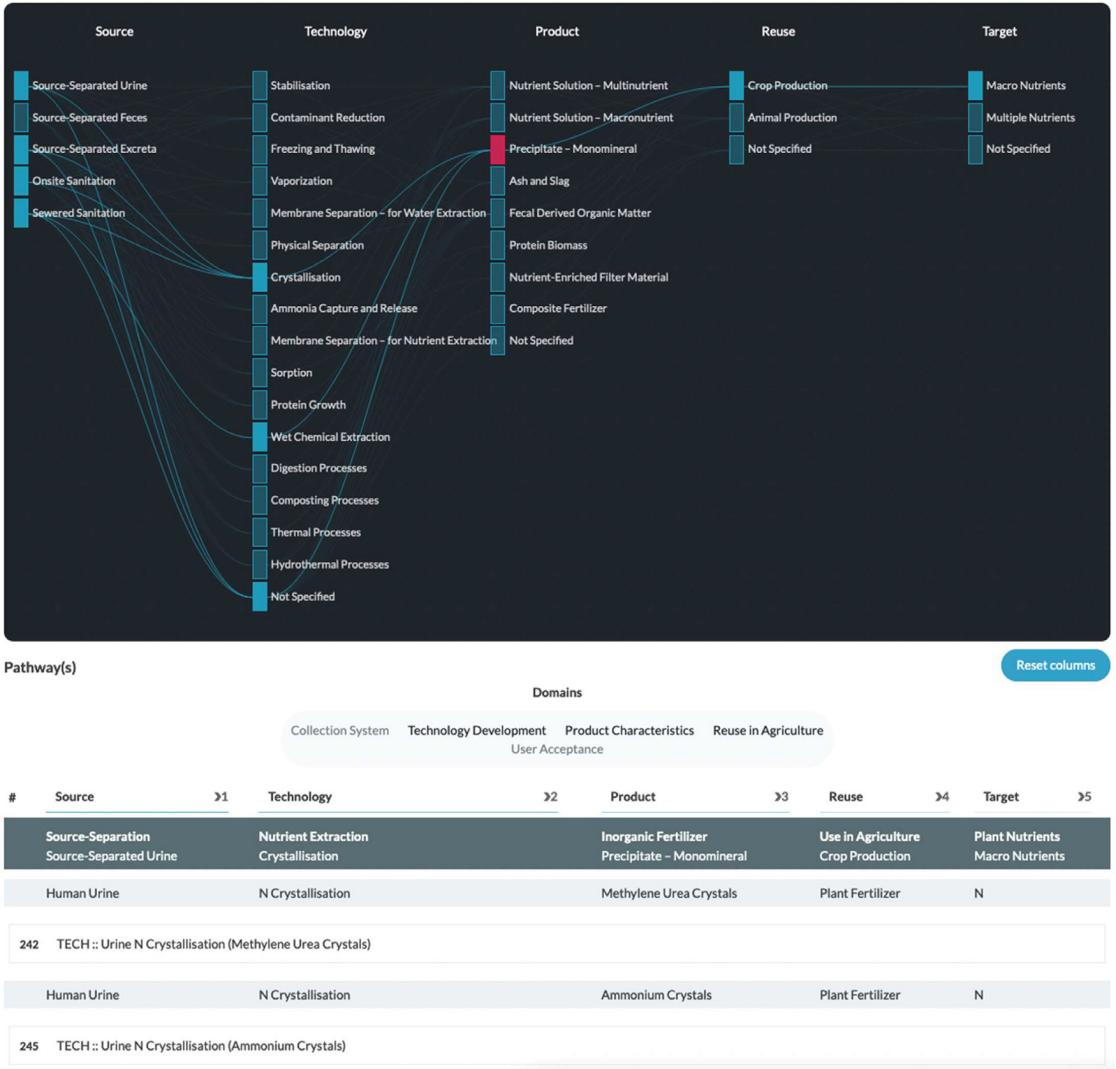
‘Option Space’ section – with activated selection for ‘Precipitate – Monomineral’. *Upper part*: option space graph. *Lower part*: associated research clusters.

***Optionspace.*** This section essentially spans the recovery pathways reported in the scientific literature. In other words, it provides an overview of investigated combinations of source, technology, product, reuse, and target. Additionally, each recovery pathway in combination with a research domain (i.e., collection system, technology development, product characteristics, use of products in agriculture, or user acceptance) represents a research cluster. Egestabase currently distinguishes 427 research clusters (346 focusing on technology development, 52 on reuse in agriculture, 21 on product characteristics, 5 on user acceptance and 3 on collection systems). We developed a visual representation to highlight connections between different recovery pathway dimensions (Fig. 5). Clicking a node in the option space graph visually shows all recovery pathways that contain this node. Underneath the option space graph, all research clusters that match the highlighted recovery pathways are shown. This makes it possible to swiftly discover and explore research clusters of interest.

**Fig. 6:**
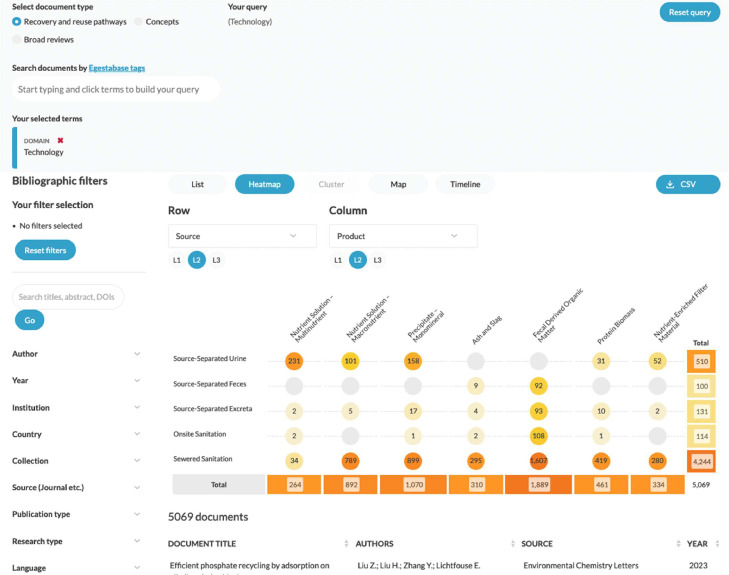
‘Research Papers’ section – with activated selection for papers on ‘technology development’. *Upper part*: searching by Egestabase tags as per the framework. *Lower part*: heatmap view across selected dimensions.

***Researchpapers.*** This section is designed to quickly find scientific publications using standardized terminology. In addition to listing relevant scientific publications, there is a map and timeline view to see the temporal and spatial distribution of research. The heatmap view allows identification research activity by means of a cross-tabulation of various recovery pathway dimensions (Fig. 6).

### Egestabase Practice

The conceptual framework for Egestabase Practice is somewhat simplified in that recovery pathways only encompass four dimensions – source, technology, product, and reuse – and only one hierarchy level. There is no separate section that outlines the framework. The four dimensions are introduced on the ‘Start’ page and options are shown in the filter part of the ‘Projects’ section.

**Fig. 7:**
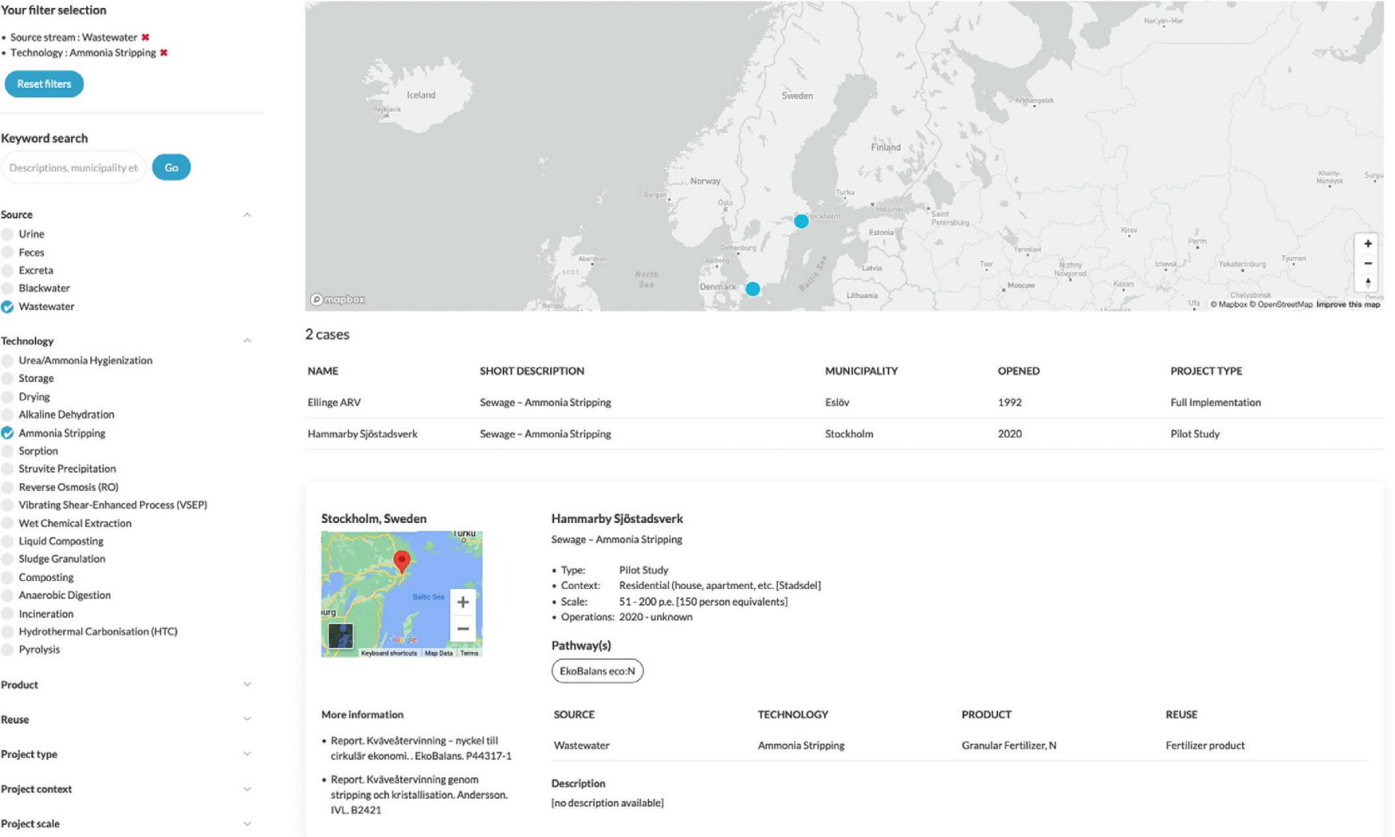
‘Projects’ section – with activated filters for ‘Wastewater’ and ‘Ammonia Stripping’. N.B. users can zoom to better see the spatial distribution of projects, or click on a project to obtain more information.

***Projects.*** This section is designed to find implementation projects and see where certain technologies have been implemented (Fig. 7).

### Outlook

***Limitations andfurther****p****otential.*** Limitations regarding the search and coding strategy for research papers are discussed in detail in Macura et al. [19]. In summary, the sheer amount of research meant we could only categorize research papers at a rather general level – recovery pathway dimensions and research domain but not more specific research details and findings. In addition to the current categorization of research papers in Egestabase Research, future work could extract additional metadata from included research papers and apply a more fine-grained categorization. For example, papers that focus on recovery technology could be further distinguished into papers about the fate of nutrients, the fate of pathogens, sustainability aspects, etc. Likewise, papers on the reuse of recovery products in agriculture could be further distinguished into papers on their agronomic value, impact on soil quality, the soil microbiome, etc. In a similar vein, specific research details could be extracted. For instance, for research on struvite precipitation, details about reagents (e.g., MgO, MgCl, seawater brine) could be extracted from the respective research papers. For research on nutrient adsorption, it would be possible to extract the type of sorbent (e.g., zeolite, biochar). Finally, apart from metadata and descriptors of studies, it would be possible to also extract and collate research findings or data from included papers (e.g. on technological effectiveness, efficiency and similar). A more fine-grained categorization and the extraction of research findings, however, would come with a major effort that by far exceeds what can realistically be achieved by a small group of researchers. Here, either artificial intelligence (AI) would need to come to the rescue (although this comes with its own challenges), or a small group of researchers could lead and coordinate a collaborative crowd-based effort where researchers from around the world contribute to categorize papers in the subject areas they are most interested in or knowledgeable about.

Evidence from practice was geographically limited to case examples from Sweden. This is in part because case examples are often poorly documented in the scientific and gray literature, in part because documentation often is in the local language. For now, the ‘Projects’ section of Egestabase Practice thus is to be seen as a proof-of-concept based on one country. A similar effort has previously been undertaken jointly by RIONED Foundation and the Dutch Foundation for Applied Water Research STOWA. Their online evidence platform ‘Saniwijzer’ (https://www.saniwijzer.nl) showcases (mostly Dutch) case examples alongside an overview of solutions and technologies (in Dutch). Other websites that highlight examples of implementation in practice include ‘Success Stories’ by the ‘Dutch Nutrient Platform’ (https://www.nutrientplatform.org/en/success-stories), ‘Projects’ by the ‘German Phosphorus Platform’ (https://www.deutsche-phosphor-plattform.de/information/projekte), and ‘Projects’ by the Swiss network for resource-oriented sanitation ‘VaLoo’ (https://va-loo.ch/de/discover/#projects). In a similar vein, the sustainable sanitation alliance ‘SUSANA’ maintains a global database of ‘Sanitation Case Studies’ (https://www.susana.org/en/knowledge-hub/resources-and-publications/case-studies), though the focus is not primarily on nutrient recovery. Regarding evidence from practice, there is thus potential for consolidation and harmonization across databases and sites.

***Future****u****pdates.*** Egestabase will remain most useful if it is being updated regularly, as this ensures that new developments are reflected in the evidence base and option space. For Egestabase Research, we believe that researchers are best suited to take care of future updates. Given current research output (around 1 000 relevant papers per year), we estimate that keeping Egestabase Research up-to-date will require about one work week per year of research output. For Egestabase Practice, we believe an organization closer to practice would be more suited than researchers to keep the implementation project database up to date. In Sweden, this could be an organization like the Swedish Nutrient Platform (Svenska Näringsplattformen) or the Swedish Water Association (Svenskt Vatten). If Egestabase Practice was to cover case studies beyond Sweden, the Sustainable Sanitation Alliance (SuSanA) might be an appropriate host for the implementation example database.

***Futureextensions.*** Egestabase could be extended in the future by covering additional source streams (e.g., food waste or animal manure) and/or additional products and reuses (e.g., volatile fatty acids for green chemistry, filler material for brick production) with associated technologies. Conceptually, such an extension could be integrated into the current framework rather easily. However, finding and categorizing the respective evidence would require several months of work.

## Conclusion

Egestabase emerged from a major collaborative effort to (a) find and categorize evidence from research and practice on nutrient recovery from human excreta and domestic wastewater for reuse in agriculture, and (b) devise an online evidence platform that allows exploration of this evidence with ease. We believe that Egestabase and its various features can be useful in a number of contexts, ranging from future research and synthesis efforts to education, and from funding decisions to resource recovery practice and implementation. More specifically, the ‘Option Space’ section – if updated regularly – could help researchers stay on top of new developments in the field as a whole. It could also be useful in an educational setting for students in environmental engineering or similar fields. In the ‘Research Papers’ section, the document view could be valuable for researchers seeking to review a specific subset of the literature, and wanting to complement their own literature search with papers not found in their own search. The heatmap and timeline views might be useful for funding agencies who may want to get a quick overview of research activities across different research clusters. The ‘Projects’ section may be useful for municipalities and utilities dealing with resource recovery, and who want to find solutions that might be of interest to them and that have already been implemented elsewhere.

## Ethics statements

Not applicable.

## CRediT authorship contribution statement

**Robin Harder** acquired funding for and led the project End-of-wastewater, created design specifications for Egestabase, screened and coded the majority of research papers included in Egestabase, and refined the implementation project database. He drafted the initial version of this manuscript. All co-authors reviewed and provided comments on successive iterations of the manuscript. **Geneviève Metson** was involved in funding acquisition, co-led actor engagement throughout the Egestabase development process, and helped quality control coding and iteratively review design choices. **Biljana Macura** was involved in funding acquisition, contributed to actor engagement throughout the Egestabase development, co-devised evidence synthesis methods, co-led the evidence synthesis process, analysed data and led the resulting associated systematic map publications. **Solveig Johannesdottir** identified implementation projects and prepared the initial version of the implementation project database. **Rosanne Wielemaker** provided key input for the major update from Egestabase R2 to R3, which covered both design and functionality of Egestabase. Dan Seddon built successive iterations of the Egestabase website based on the design specifications provided by the team. **Abdulhamid Aliahmad** helped coding conceptual papers. Emma Lundin contributed to stakeholder mapping and co-organised early stakeholder engagement. **Erik Kärrman** supported the identification of implementation projects in a supervisory role. **Jennifer McConville** was involved in funding acquisition, co-led actor engagement throughout the Egestabase development process, helped quality control coding, and iteratively reviewed design specifications and coding terminology.

## Declaration of competing interest

The authors declare that they have no known competing financial interests or personal relationships that could have appeared to influence the work reported in this paper.

## Data Availability

Data will be made available on request.

## Footnotes

1 The term ‘egesta’ refers to matter that is digested from the body, as excrement or other waste. Our use of the term is inspired by the Egesta Lab at the University of British Columbia. As suggested by Dr. Gunilla Öberg of the Egesta Lab, the term ‘egesta’ is not tied to a certain way of thinking and thus invites us to re-frame challenges and opportunities as well as re-imagine workable solutions.
